# 

**Published:** 1858-10

**Authors:** 


### CONTENTS.

ORIGINAL COMMUNICATIONS— PAGE.

On some of the Causes of Puerperal Convulsions. By Ralph N. Isham, M. D. .... 485

Strangulated Hernia, etc. By William Dickinson, M.D .... 497

Cases in Practice. By Thos. W. Fry, M.D .... 503

A Case of Anchylosis of both Elbow Joints, treated by Forced Rupture of the Adhesions. By J. W. Freer, M. D .... 509

A Case of Inversio Uteri, occurring without the usual Symptoms. By A. Fisher, M. D .... 510

The Use of Plates, in combination with Gutta Percha, in Treatment of Fractures of the Lower Jaw. By W. W. Allport, Dentist .... 514

A New Mode of Dressing Fractured Clavicle. By J. W. Freer, M.D. .... 517

Report of the Committee on Practical Medicine. By F. K. Bailey, M.D. 519

BOOK AND PAMPHLET NOTICES—

A Manual of the Practice of Medicine. By T. H. Tanner, M. D. .... 533

Transactions of the New Hampshire Medical Society .... 533

An Essay on the Pathology and Therapeutics of Scarlet Fever. By Casper Morris, M.D .... 535

The Belmont Medical Journal .... 535

EDITORIAL—

To Subscribers .... 536

Inversion of the Uterus .... 536

Clinic at the Mercy Hospital, by Prof. N. S. Davis. Reported by E. A. Steele, A.B. .... 538

Sixteenth Annual Course of Lectures in Rush Medical College .... 543

Professional Appointments .... 544

New York State Inebriate Asylum .... 544

